# Crystal structure of di-*n*-but­yl­bis­(*η* ^5^-penta­methyl­cyclo­penta­dien­yl)hafnium(IV)

**DOI:** 10.1107/S2056989015000092

**Published:** 2015-01-10

**Authors:** Perdita Arndt, Kathleen Schubert, Vladimir V. Burlakov, Anke Spannenberg, Uwe Rosenthal

**Affiliations:** a Leibniz-Institut für Katalyse e. V. an der Universität Rostock, Albert-Einstein-Str. 29a, 18059 Rostock, Germany; b A. N. Nesmeyanov Institute of Organoelement Compounds, Russian Academy of Sciences, Vavilov St 28, 119991, Moscow, Russian Federation

**Keywords:** crystal structure, hafnocene, *n*-but­yl, racemic twin

## Abstract

The crystal structure of the title compound, [Hf(C_10_H_15_)_2_(C_4_H_9_)_2_], reveals two independent mol­ecules in the asymmetric unit. The diffraction experiment was performed with a racemically twinned crystal showing a 0.529 (5):0.471 (5) component ratio. Each Hf^IV^ atom is coordinated by two penta­methyl­cyclo­penta­dienyl and two *n*-butyl ligands in a distorted tetra­hedral geometry, with the cyclo­penta­dienyl rings inclined to one another by 45.11 (15) and 45.37 (16)°. In contrast to the isostructural di(*n*-butyl)bis(*η* ^5^-penta­methyl­cyclo­penta­dien­yl)zirconium(IV) complex with a noticeable difference in the Zr–butyl bonding, the Hf—C_but­yl_ bond lengths differ from each other by no more than 0.039 (3) Å.

## Related literature   

For the synthesis of the title compound, see: Schock & Marks (1988 ▸), and for that of the corresponding unsubstituted cyclo­penta­dienyl complex, see: Burlakov *et al.* (2008 ▸). For the use of these complexes as the starting materials for various reactions, see: Burlakov *et al.* (2009 ▸). For the structure of the isostructural zirconocene complex, see: Ernst *et al.* (2004 ▸), and of an *ansa*-zirconocene with an additional Zr—N bond, see: Paolucci *et al.* (1997 ▸). For [Mo(C_5_H_5_)_2_(C_4_H_9_)_2_], see: Calhorda *et al.* (1991 ▸).
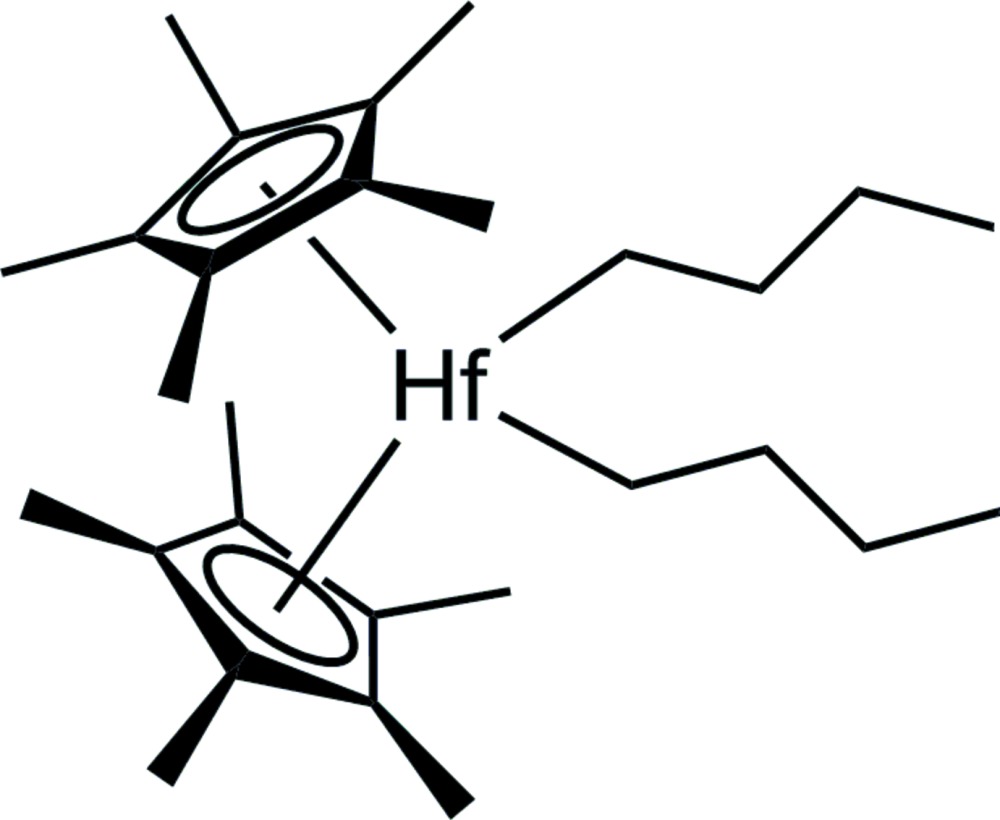


## Experimental   

### Crystal data  

[Hf(C_10_H_15_)_2_(C_4_H_9_)_2_]
*M* *_r_* = 563.15
Orthorhombic, 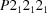

*a* = 17.1572 (3) Å
*b* = 17.2320 (3) Å
*c* = 17.2966 (3) Å
*V* = 5113.79 (15) Å^3^
*Z* = 8
Mo *K*α radiation
μ = 4.09 mm^−1^
*T* = 170 K
0.39 × 0.27 × 0.17 mm

### Data collection  

Bruker Kappa APEXII DUO CCD diffractometer
Absorption correction: multi-scan (*SADABS*; Bruker, 2008 ▸) *T*_min_ = 0.30, *T*_max_ = 0.54
123798 measured reflections
13236 independent reflections
12433 reflections with *I* > 2σ(*I*)
*R*_int_ = 0.037

### Refinement  

*R*[*F* ^2^ > 2σ(*F* ^2^)] = 0.018
*wR*(*F* ^2^) = 0.038
*S* = 1.06
13236 reflections
548 parameters
H-atom parameters constrained
Δρ_max_ = 0.53 e Å^−3^
Δρ_min_ = −0.77 e Å^−3^
Absolute structure: Flack (1983 ▸), 999 Friedel pairs
Absolute structure parameter: 0.471 (5)

### 

Data collection: *APEX2* (Bruker, 2008 ▸); cell refinement: *SAINT* (Bruker, 2008 ▸); data reduction: *SAINT*; program(s) used to solve structure: *SHELXS97* (Sheldrick, 2008 ▸); program(s) used to refine structure: *SHELXL2014* (Sheldrick, 2008 ▸); molecular graphics: *XP* in *SHELXTL* (Sheldrick, 2008 ▸); software used to prepare material for publication: *SHELXL2014*.

## Supplementary Material

Crystal structure: contains datablock(s) I, New_Global_Publ_Block. DOI: 10.1107/S2056989015000092/yk2105sup1.cif

Structure factors: contains datablock(s) I. DOI: 10.1107/S2056989015000092/yk2105Isup2.hkl

**Fig. 1.**
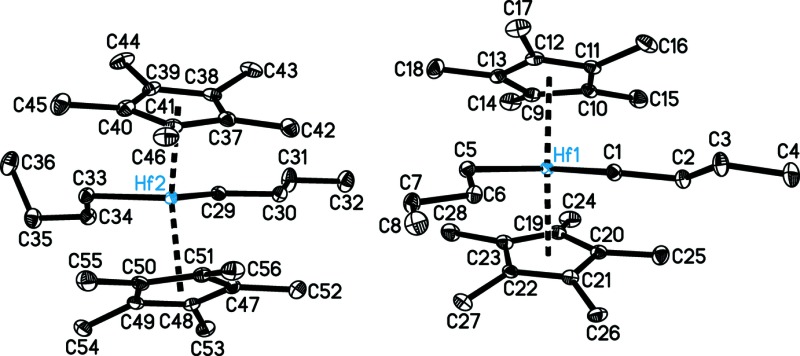
: Molecular structure of the two molecules of the asymmetric unit with labelling and displacement ellipsoids drawn at 30% probability level. Hydrogen atoms are omitted for clarity.

. DOI: 10.1107/S2056989015000092/yk2105fig1.tif
Mol­ecular structure of the two mol­ecules of the asymmetric unit with labelling and displacement ellipsoids drawn at 30% probability level. Hydrogen atoms are omitted for clarity.

CCDC reference: 1042126

Additional supporting information:  crystallographic information; 3D view; checkCIF report

## supplementary crystallographic information

### S1. Synthesis and crystallization

The title compound was synthesized as described by Schock & Marks (1988). To a suspension of Cp*_2_HfCl_2_ (1.00 g, 1.94 mmol) in 20 ml of di­ethyl­ether were added 3.0 ml of *n*-BuLi (1.6 *M*, 4.8 mmol) in di­ethyl­ether. The solution was stirred for 3 days at RT before the ether was removed *in vacuo*. The residue was extracted with *n*-hexane three times, the collected filtrates were reduced *in vacuo* to 10 ml. At -78°C colorless crystals were formed within two weeks (0,545 g, yield 50%).

^1^H NMR (300 MHz, C_6_D_6_): δ = - 0.14 (t, 4H, α-CH_2_), 1.05 (t, 6H, CH_3_), 1.23 (m, 4H, γ-CH_2_), 1.44 (t, 4H β-CH_2_), 1.84 (s, 30 H, *Me*-Cp) ppm., see Schock & Marks (1988).

### S2. Refinement

H atoms were placed in idealized positions with d(C—H) = 0.99 Å (CH_2_), 0.98 Å (CH_3_) and refined using a riding model with *U*_iso_(H) fixed at 1.2 *U*_eq_(C) for CH_2_ and 1.5 *U*_eq_(C) for CH_3_. The title compound crystallizes as racemic twin (Flack parameter x = 0.471 (5)).

### Crystal data

**Table:**

[Hf(C_10_H_15_)_2_(C_4_H_9_)_2_]	*D*_x_ = 1.463 Mg m^−^^3^
*M**_r_* = 563.15	Mo *K*α radiation, λ = 0.71073 Å
Orthorhombic, *P*2_1_2_1_2_1_	Cell parameters from 9300 reflections
*a* = 17.1572 (3) Å	θ = 2.4–28.7°
*b* = 17.2320 (3) Å	µ = 4.09 mm^−^^1^
*c* = 17.2966 (3) Å	*T* = 170 K
*V* = 5113.79 (15) Å^3^	Prism, colourless
*Z* = 8	0.39 × 0.27 × 0.17 mm
*F*(000) = 2304	

### Data collection

**Table:**

Bruker Kappa APEXII DUO CCD diffractometer	13236 independent reflections
Radiation source: fine-focus sealed tube	12433 reflections with *I* > 2σ(*I*)
Curved graphite monochromator	*R*_int_ = 0.037
Detector resolution: 8.3333 pixels mm^-1^	θ_max_ = 28.7°, θ_min_ = 1.7°
φ and ω scans	*h* = −18→23
Absorption correction: multi-scan (*SADABS*; Bruker, 2008)	*k* = −23→23
*T*_min_ = 0.30, *T*_max_ = 0.54	*l* = −23→23
123798 measured reflections	

### Refinement

**Table:**

Refinement on *F*^2^	Secondary atom site location: difference Fourier map
Least-squares matrix: full	Hydrogen site location: inferred from neighbouring sites
*R*[*F*^2^ > 2σ(*F*^2^)] = 0.018	H-atom parameters constrained
*wR*(*F*^2^) = 0.038	*w* = 1/[σ^2^(*F*_o_^2^) + (0.017*P*)^2^ + 1.9262*P*] where *P* = (*F*_o_^2^ + 2*F*_c_^2^)/3
*S* = 1.06	(Δ/σ)_max_ = 0.003
13236 reflections	Δρ_max_ = 0.53 e Å^−^^3^
548 parameters	Δρ_min_ = −0.77 e Å^−^^3^
0 restraints	Absolute structure: Flack (1983), 999 Friedel pairs
Primary atom site location: structure-invariant direct methods	Absolute structure parameter: 0.471 (5)

### Special details

**Table:**

Geometry. All e.s.d.'s (except the e.s.d. in the dihedral angle between two l.s. planes) are estimated using the full covariance matrix. The cell e.s.d.'s are taken into account individually in the estimation of e.s.d.'s in distances, angles and torsion angles; correlations between e.s.d.'s in cell parameters are only used when they are defined by crystal symmetry. An approximate (isotropic) treatment of cell e.s.d.'s is used for estimating e.s.d.'s involving l.s. planes.
Refinement. Refinement of *F*^2^ against ALL reflections. The weighted *R*-factor *wR* and goodness of fit *S* are based on *F*^2^, conventional *R*-factors *R* are based on *F*, with *F* set to zero for negative *F*^2^. The threshold expression of *F*^2^ > σ(*F*^2^) is used only for calculating *R*-factors(gt) *etc*. and is not relevant to the choice of reflections for refinement. *R*-factors based on *F*^2^ are statistically about twice as large as those based on *F*, and *R*- factors based on ALL data will be even larger.

### Fractional atomic coordinates and isotropic or equivalent isotropic displacement parameters (Å^2^)

**Table:**

	*x*	*y*	*z*	*U*_iso_*/*U*_eq_	
C1	0.10245 (14)	1.04413 (14)	0.84536 (16)	0.0209 (5)	
H1A	0.0775	1.0294	0.7959	0.025*	
H1B	0.0711	1.0192	0.8865	0.025*	
C2	0.08748 (16)	1.13134 (15)	0.85402 (17)	0.0234 (6)	
H2A	0.0962	1.1463	0.9086	0.028*	
H2B	0.1257	1.1599	0.8220	0.028*	
C3	0.00596 (16)	1.15583 (16)	0.83060 (19)	0.0300 (7)	
H3A	−0.0322	1.1245	0.8602	0.036*	
H3B	−0.0015	1.1438	0.7751	0.036*	
C4	−0.0116 (2)	1.24120 (17)	0.8436 (2)	0.0377 (8)	
H4A	0.0234	1.2728	0.8118	0.057*	
H4B	−0.0039	1.2540	0.8983	0.057*	
H4C	−0.0658	1.2519	0.8291	0.057*	
C5	0.16087 (15)	0.86169 (15)	0.84278 (16)	0.0227 (5)	
H5A	0.1638	0.8424	0.7889	0.027*	
H5B	0.1925	0.8262	0.8750	0.027*	
C6	0.07551 (15)	0.85520 (15)	0.86963 (16)	0.0211 (5)	
H6A	0.0699	0.8814	0.9202	0.025*	
H6B	0.0416	0.8825	0.8321	0.025*	
C7	0.04807 (17)	0.77125 (16)	0.87731 (18)	0.0260 (6)	
H7A	0.0595	0.7435	0.8285	0.031*	
H7B	0.0781	0.7458	0.9191	0.031*	
C8	−0.03837 (18)	0.76363 (18)	0.8950 (2)	0.0358 (7)	
H8A	−0.0687	0.7876	0.8533	0.054*	
H8B	−0.0500	0.7898	0.9440	0.054*	
H8C	−0.0521	0.7086	0.8991	0.054*	
C9	0.32937 (14)	0.98398 (18)	0.74945 (14)	0.0213 (5)	
C10	0.29051 (18)	1.05690 (15)	0.74342 (14)	0.0211 (5)	
C11	0.21592 (19)	1.04259 (13)	0.71054 (13)	0.0211 (5)	
C12	0.20807 (17)	0.96162 (15)	0.69862 (14)	0.0227 (5)	
C13	0.27865 (19)	0.92560 (15)	0.72268 (14)	0.0232 (5)	
C14	0.41568 (15)	0.9711 (2)	0.76112 (17)	0.0330 (7)	
H14A	0.4400	0.9582	0.7115	0.049*	
H14B	0.4236	0.9282	0.7976	0.049*	
H14C	0.4394	1.0184	0.7819	0.049*	
C15	0.32455 (19)	1.13609 (18)	0.75601 (19)	0.0328 (7)	
H15A	0.2975	1.1616	0.7990	0.049*	
H15B	0.3183	1.1671	0.7089	0.049*	
H15C	0.3801	1.1313	0.7683	0.049*	
C16	0.16141 (18)	1.10256 (19)	0.67941 (18)	0.0342 (7)	
H16A	0.1647	1.1033	0.6228	0.051*	
H16B	0.1758	1.1536	0.6998	0.051*	
H16C	0.1080	1.0900	0.6951	0.051*	
C17	0.13977 (18)	0.9248 (2)	0.65925 (18)	0.0360 (7)	
H17A	0.1460	0.8682	0.6596	0.054*	
H17B	0.1368	0.9432	0.6057	0.054*	
H17C	0.0918	0.9387	0.6867	0.054*	
C18	0.29965 (19)	0.84198 (17)	0.71220 (19)	0.0365 (8)	
H18A	0.3252	0.8227	0.7591	0.055*	
H18B	0.3353	0.8367	0.6682	0.055*	
H18C	0.2523	0.8117	0.7024	0.055*	
C19	0.32414 (14)	1.02052 (19)	0.94309 (14)	0.0225 (5)	
C20	0.25657 (15)	1.06311 (16)	0.96461 (15)	0.0212 (5)	
C21	0.20008 (14)	1.00970 (16)	0.99217 (15)	0.0220 (6)	
C22	0.23236 (15)	0.93384 (15)	0.98649 (14)	0.0203 (6)	
C23	0.30815 (15)	0.94104 (17)	0.95437 (16)	0.0225 (6)	
C24	0.40301 (17)	1.05688 (19)	0.93168 (18)	0.0334 (7)	
H24A	0.4402	1.0171	0.9148	0.050*	
H24B	0.4208	1.0796	0.9805	0.050*	
H24C	0.3995	1.0976	0.8923	0.050*	
C25	0.25347 (18)	1.14995 (16)	0.97023 (19)	0.0315 (7)	
H25A	0.2059	1.1655	0.9975	0.047*	
H25B	0.2534	1.1724	0.9182	0.047*	
H25C	0.2991	1.1687	0.9987	0.047*	
C26	0.12593 (15)	1.02885 (19)	1.03341 (15)	0.0274 (6)	
H26A	0.1121	1.0831	1.0236	0.041*	
H26B	0.1330	1.0209	1.0891	0.041*	
H26C	0.0841	0.9950	1.0146	0.041*	
C27	0.19842 (17)	0.86248 (17)	1.02273 (17)	0.0300 (7)	
H27A	0.1468	0.8524	1.0006	0.045*	
H27B	0.1936	0.8703	1.0786	0.045*	
H27C	0.2327	0.8181	1.0127	0.045*	
C28	0.36408 (18)	0.87419 (18)	0.94451 (18)	0.0330 (7)	
H28A	0.4141	0.8937	0.9254	0.049*	
H28B	0.3425	0.8370	0.9073	0.049*	
H28C	0.3719	0.8484	0.9944	0.049*	
C29	0.39487 (15)	0.54682 (14)	0.88712 (16)	0.0206 (5)	
H29A	0.4240	0.5259	0.9319	0.025*	
H29B	0.4238	0.5293	0.8408	0.025*	
C30	0.40920 (15)	0.63443 (15)	0.89038 (17)	0.0229 (5)	
H30A	0.3763	0.6603	0.8511	0.028*	
H30B	0.3934	0.6541	0.9418	0.028*	
C31	0.49422 (17)	0.65535 (17)	0.8760 (2)	0.0368 (8)	
H31A	0.5268	0.6278	0.9145	0.044*	
H31B	0.5092	0.6361	0.8242	0.044*	
C32	0.5129 (2)	0.74127 (17)	0.8802 (2)	0.0395 (8)	
H32A	0.4989	0.7612	0.9315	0.059*	
H32B	0.4832	0.7691	0.8406	0.059*	
H32C	0.5688	0.7491	0.8714	0.059*	
C33	0.35133 (16)	0.36285 (15)	0.88524 (16)	0.0230 (5)	
H33A	0.3197	0.3240	0.9134	0.028*	
H33B	0.3559	0.3447	0.8311	0.028*	
C34	0.43333 (16)	0.36327 (16)	0.92074 (17)	0.0257 (6)	
H34A	0.4666	0.3985	0.8898	0.031*	
H34B	0.4299	0.3851	0.9736	0.031*	
C35	0.47354 (18)	0.28392 (17)	0.9256 (2)	0.0334 (7)	
H35A	0.5219	0.2892	0.9564	0.040*	
H35B	0.4388	0.2472	0.9530	0.040*	
C36	0.4937 (2)	0.25057 (19)	0.8475 (2)	0.0411 (8)	
H36A	0.5313	0.2846	0.8215	0.062*	
H36B	0.4464	0.2466	0.8160	0.062*	
H36C	0.5166	0.1989	0.8541	0.062*	
C37	0.2159 (2)	0.55026 (15)	0.77661 (14)	0.0228 (5)	
C38	0.29141 (17)	0.53422 (15)	0.74613 (13)	0.0220 (6)	
C39	0.29843 (15)	0.45287 (16)	0.73771 (15)	0.0227 (6)	
C40	0.22729 (18)	0.41820 (16)	0.76189 (16)	0.0241 (6)	
C41	0.17574 (14)	0.47824 (19)	0.78436 (14)	0.0225 (5)	
C42	0.18260 (18)	0.63014 (17)	0.78753 (18)	0.0305 (7)	
H42A	0.1660	0.6509	0.7374	0.046*	
H42B	0.2224	0.6642	0.8099	0.046*	
H42C	0.1377	0.6274	0.8224	0.046*	
C43	0.34800 (19)	0.59149 (19)	0.71299 (17)	0.0331 (7)	
H43A	0.3474	0.5879	0.6565	0.050*	
H43B	0.4005	0.5800	0.7321	0.050*	
H43C	0.3331	0.6441	0.7287	0.050*	
C44	0.36646 (18)	0.4137 (2)	0.70034 (19)	0.0387 (8)	
H44A	0.4142	0.4271	0.7284	0.058*	
H44B	0.3709	0.4311	0.6465	0.058*	
H44C	0.3589	0.3574	0.7016	0.058*	
C45	0.2069 (2)	0.33404 (17)	0.75571 (19)	0.0361 (7)	
H45A	0.1969	0.3209	0.7015	0.054*	
H45B	0.1601	0.3234	0.7865	0.054*	
H45C	0.2503	0.3026	0.7751	0.054*	
C46	0.08939 (16)	0.4674 (2)	0.79405 (17)	0.0331 (7)	
H46A	0.0796	0.4268	0.8325	0.050*	
H46B	0.0664	0.4521	0.7445	0.050*	
H46C	0.0659	0.5162	0.8115	0.050*	
C47	0.23728 (15)	0.55693 (16)	1.00118 (15)	0.0209 (5)	
C48	0.29283 (17)	0.50411 (14)	1.03097 (14)	0.0218 (6)	
C49	0.26283 (16)	0.42810 (16)	1.02337 (15)	0.0230 (6)	
C50	0.18791 (16)	0.43383 (17)	0.98829 (16)	0.0221 (6)	
C51	0.17114 (15)	0.51383 (18)	0.97676 (15)	0.0222 (5)	
C52	0.23970 (17)	0.64415 (16)	1.00693 (18)	0.0308 (7)	
H52A	0.2876	0.6601	1.0334	0.046*	
H52B	0.1944	0.6625	1.0363	0.046*	
H52C	0.2387	0.6666	0.9549	0.046*	
C53	0.36538 (15)	0.52551 (18)	1.07501 (16)	0.0293 (6)	
H53A	0.4056	0.4862	1.0660	0.044*	
H53B	0.3535	0.5281	1.1304	0.044*	
H53C	0.3843	0.5762	1.0573	0.044*	
C54	0.29470 (18)	0.35685 (16)	1.06056 (16)	0.0299 (6)	
H54A	0.2809	0.3566	1.1155	0.045*	
H54B	0.3516	0.3562	1.0552	0.045*	
H54C	0.2727	0.3108	1.0354	0.045*	
C55	0.13305 (19)	0.36651 (18)	0.97753 (19)	0.0355 (7)	
H55A	0.0846	0.3849	0.9539	0.053*	
H55B	0.1215	0.3431	1.0279	0.053*	
H55C	0.1574	0.3277	0.9439	0.053*	
C56	0.09175 (17)	0.54926 (19)	0.96328 (18)	0.0330 (7)	
H56A	0.0955	0.5892	0.9231	0.049*	
H56B	0.0729	0.5727	1.0114	0.049*	
H56C	0.0553	0.5088	0.9466	0.049*	
Hf1	0.218019 (6)	0.981329 (5)	0.846352 (5)	0.01504 (2)	
Hf2	0.283647 (6)	0.477447 (5)	0.884701 (5)	0.01551 (2)	

### Atomic displacement parameters (Å^2^)

**Table:**

	*U*^11^	*U*^22^	*U*^33^	*U*^12^	*U*^13^	*U*^23^
C1	0.0178 (12)	0.0213 (12)	0.0235 (13)	0.0033 (9)	0.0016 (10)	0.0020 (10)
C2	0.0213 (14)	0.0206 (13)	0.0282 (15)	0.0014 (10)	−0.0010 (11)	0.0015 (11)
C3	0.0211 (14)	0.0229 (14)	0.0461 (19)	0.0044 (11)	−0.0013 (13)	0.0031 (12)
C4	0.0388 (19)	0.0273 (15)	0.047 (2)	0.0118 (13)	−0.0021 (16)	0.0025 (14)
C5	0.0247 (14)	0.0205 (12)	0.0228 (13)	−0.0034 (10)	0.0026 (11)	−0.0011 (10)
C6	0.0182 (13)	0.0200 (12)	0.0253 (14)	−0.0014 (10)	−0.0015 (10)	−0.0014 (10)
C7	0.0260 (15)	0.0201 (12)	0.0320 (15)	−0.0040 (11)	0.0008 (12)	−0.0015 (12)
C8	0.0285 (16)	0.0370 (16)	0.0421 (19)	−0.0115 (13)	0.0016 (13)	−0.0002 (14)
C9	0.0154 (12)	0.0299 (13)	0.0184 (11)	0.0004 (12)	0.0061 (9)	0.0006 (12)
C10	0.0201 (14)	0.0246 (12)	0.0187 (12)	0.0008 (12)	0.0033 (11)	0.0057 (10)
C11	0.0186 (11)	0.0284 (13)	0.0163 (10)	0.0020 (13)	0.0021 (11)	0.0043 (9)
C12	0.0212 (14)	0.0308 (14)	0.0162 (11)	−0.0028 (11)	0.0019 (10)	−0.0011 (9)
C13	0.0249 (13)	0.0266 (12)	0.0182 (11)	0.0042 (13)	0.0074 (12)	−0.0006 (9)
C14	0.0167 (13)	0.0515 (19)	0.0309 (15)	0.0051 (14)	0.0056 (11)	0.0018 (15)
C15	0.0354 (17)	0.0306 (16)	0.0323 (17)	−0.0080 (13)	0.0027 (13)	0.0057 (13)
C16	0.0277 (16)	0.0476 (18)	0.0272 (16)	0.0102 (14)	0.0030 (12)	0.0139 (14)
C17	0.0293 (16)	0.052 (2)	0.0266 (16)	−0.0082 (14)	0.0015 (13)	−0.0051 (14)
C18	0.042 (2)	0.0298 (15)	0.0374 (17)	0.0064 (13)	0.0169 (14)	−0.0036 (12)
C19	0.0205 (12)	0.0292 (13)	0.0177 (12)	−0.0007 (12)	−0.0047 (9)	0.0012 (12)
C20	0.0237 (13)	0.0228 (13)	0.0172 (12)	−0.0055 (10)	−0.0026 (10)	−0.0014 (11)
C21	0.0211 (15)	0.0256 (13)	0.0192 (11)	0.0000 (10)	0.0003 (9)	0.0012 (10)
C22	0.0223 (16)	0.0237 (12)	0.0149 (11)	0.0030 (10)	−0.0017 (10)	0.0026 (10)
C23	0.0176 (13)	0.0303 (14)	0.0196 (13)	0.0046 (11)	−0.0046 (10)	0.0034 (11)
C24	0.0257 (15)	0.0470 (17)	0.0275 (15)	−0.0080 (13)	0.0011 (12)	0.0012 (14)
C25	0.0351 (16)	0.0253 (14)	0.0341 (16)	−0.0023 (12)	−0.0048 (13)	−0.0049 (13)
C26	0.0275 (14)	0.0367 (16)	0.0180 (12)	0.0036 (13)	0.0044 (10)	−0.0022 (12)
C27	0.0330 (18)	0.0307 (14)	0.0262 (14)	−0.0011 (12)	0.0005 (12)	0.0083 (12)
C28	0.0283 (16)	0.0400 (17)	0.0306 (17)	0.0106 (13)	−0.0013 (12)	0.0056 (13)
C29	0.0203 (13)	0.0224 (12)	0.0190 (12)	−0.0028 (9)	−0.0017 (10)	0.0002 (10)
C30	0.0205 (13)	0.0228 (13)	0.0255 (14)	−0.0014 (10)	0.0029 (11)	−0.0019 (11)
C31	0.0221 (15)	0.0233 (14)	0.065 (2)	−0.0049 (11)	0.0082 (15)	−0.0028 (14)
C32	0.0367 (19)	0.0283 (16)	0.054 (2)	−0.0094 (14)	0.0142 (16)	−0.0030 (16)
C33	0.0229 (13)	0.0222 (12)	0.0237 (13)	0.0025 (10)	−0.0013 (11)	−0.0024 (11)
C34	0.0251 (14)	0.0254 (13)	0.0267 (14)	0.0019 (11)	−0.0019 (11)	−0.0012 (12)
C35	0.0308 (16)	0.0292 (15)	0.0404 (17)	0.0067 (12)	−0.0054 (14)	0.0024 (13)
C36	0.0299 (18)	0.0337 (17)	0.060 (2)	0.0034 (13)	0.0035 (17)	−0.0128 (15)
C37	0.0228 (13)	0.0270 (12)	0.0186 (11)	0.0028 (13)	−0.0065 (13)	0.0026 (9)
C38	0.0195 (13)	0.0318 (14)	0.0147 (10)	−0.0019 (12)	−0.0026 (10)	0.0036 (10)
C39	0.0214 (16)	0.0298 (14)	0.0168 (11)	0.0043 (10)	−0.0029 (10)	−0.0069 (10)
C40	0.0204 (15)	0.0301 (14)	0.0219 (12)	−0.0012 (12)	−0.0085 (12)	−0.0040 (10)
C41	0.0195 (12)	0.0298 (13)	0.0182 (12)	0.0013 (12)	−0.0046 (9)	0.0013 (12)
C42	0.0312 (16)	0.0313 (16)	0.0289 (16)	0.0069 (12)	−0.0045 (13)	0.0040 (12)
C43	0.0319 (16)	0.0455 (18)	0.0217 (14)	−0.0090 (14)	−0.0019 (12)	0.0091 (13)
C44	0.0329 (18)	0.058 (2)	0.0251 (16)	0.0102 (15)	0.0009 (13)	−0.0147 (15)
C45	0.0346 (19)	0.0340 (15)	0.0397 (17)	−0.0056 (14)	−0.0096 (15)	−0.0088 (13)
C46	0.0222 (14)	0.051 (2)	0.0265 (14)	−0.0042 (13)	−0.0032 (11)	−0.0011 (14)
C47	0.0236 (15)	0.0219 (12)	0.0171 (12)	−0.0018 (10)	0.0040 (10)	−0.0026 (10)
C48	0.0253 (15)	0.0260 (13)	0.0142 (10)	−0.0001 (10)	0.0009 (10)	−0.0033 (9)
C49	0.0287 (17)	0.0255 (13)	0.0149 (12)	−0.0028 (11)	0.0011 (10)	0.0020 (10)
C50	0.0233 (14)	0.0259 (14)	0.0172 (13)	−0.0041 (11)	0.0037 (10)	0.0028 (11)
C51	0.0197 (12)	0.0283 (14)	0.0187 (12)	0.0012 (12)	0.0037 (9)	−0.0017 (12)
C52	0.0359 (18)	0.0273 (14)	0.0294 (15)	0.0046 (12)	0.0036 (12)	−0.0029 (12)
C53	0.0293 (14)	0.0351 (15)	0.0235 (13)	0.0000 (13)	−0.0042 (11)	−0.0049 (14)
C54	0.0364 (18)	0.0285 (13)	0.0249 (14)	0.0020 (12)	0.0018 (12)	0.0061 (11)
C55	0.0353 (18)	0.0373 (17)	0.0337 (18)	−0.0143 (14)	0.0021 (14)	0.0019 (14)
C56	0.0235 (15)	0.0464 (18)	0.0289 (15)	0.0084 (13)	0.0042 (12)	−0.0011 (13)
Hf1	0.01256 (4)	0.01658 (4)	0.01597 (4)	0.00068 (5)	0.00096 (4)	0.00147 (4)
Hf2	0.01422 (4)	0.01740 (4)	0.01492 (4)	−0.00044 (6)	−0.00092 (4)	−0.00109 (4)

### Geometric parameters (Å, º)

**Table:**

C1—C2	1.532 (3)	C29—C30	1.531 (3)
C1—Hf1	2.259 (2)	C29—Hf2	2.252 (2)
C1—H1A	0.9900	C29—H29A	0.9900
C1—H1B	0.9900	C29—H29B	0.9900
C2—C3	1.516 (4)	C30—C31	1.523 (4)
C2—H2A	0.9900	C30—H30A	0.9900
C2—H2B	0.9900	C30—H30B	0.9900
C3—C4	1.518 (4)	C31—C32	1.517 (4)
C3—H3A	0.9900	C31—H31A	0.9900
C3—H3B	0.9900	C31—H31B	0.9900
C4—H4A	0.9800	C32—H32A	0.9800
C4—H4B	0.9800	C32—H32B	0.9800
C4—H4C	0.9800	C32—H32C	0.9800
C5—C6	1.540 (4)	C33—C34	1.535 (4)
C5—Hf1	2.284 (2)	C33—Hf2	2.291 (3)
C5—H5A	0.9900	C33—H33A	0.9900
C5—H5B	0.9900	C33—H33B	0.9900
C6—C7	1.527 (4)	C34—C35	1.534 (4)
C6—H6A	0.9900	C34—H34A	0.9900
C6—H6B	0.9900	C34—H34B	0.9900
C7—C8	1.520 (4)	C35—C36	1.509 (5)
C7—H7A	0.9900	C35—H35A	0.9900
C7—H7B	0.9900	C35—H35B	0.9900
C8—H8A	0.9800	C36—H36A	0.9800
C8—H8B	0.9800	C36—H36B	0.9800
C8—H8C	0.9800	C36—H36C	0.9800
C9—C13	1.408 (4)	C37—C38	1.425 (4)
C9—C10	1.426 (4)	C37—C41	1.426 (4)
C9—C14	1.511 (3)	C37—C42	1.502 (4)
C9—Hf1	2.542 (2)	C37—Hf2	2.534 (3)
C10—C11	1.422 (4)	C38—C39	1.415 (4)
C10—C15	1.500 (4)	C38—C43	1.498 (4)
C10—Hf1	2.532 (3)	C38—Hf2	2.592 (2)
C11—C12	1.417 (4)	C39—C40	1.422 (4)
C11—C16	1.494 (4)	C39—C44	1.495 (4)
C11—Hf1	2.576 (2)	C39—Hf2	2.590 (3)
C12—C13	1.423 (4)	C40—C41	1.416 (4)
C12—C17	1.497 (4)	C40—C45	1.496 (4)
C12—Hf1	2.583 (2)	C40—Hf2	2.547 (3)
C13—C18	1.496 (4)	C41—C46	1.503 (4)
C13—Hf1	2.565 (2)	C41—Hf2	2.538 (2)
C14—H14A	0.9800	C42—H42A	0.9800
C14—H14B	0.9800	C42—H42B	0.9800
C14—H14C	0.9800	C42—H42C	0.9800
C15—H15A	0.9800	C43—H43A	0.9800
C15—H15B	0.9800	C43—H43B	0.9800
C15—H15C	0.9800	C43—H43C	0.9800
C16—H16A	0.9800	C44—H44A	0.9800
C16—H16B	0.9800	C44—H44B	0.9800
C16—H16C	0.9800	C44—H44C	0.9800
C17—H17A	0.9800	C45—H45A	0.9800
C17—H17B	0.9800	C45—H45B	0.9800
C17—H17C	0.9800	C45—H45C	0.9800
C18—H18A	0.9800	C46—H46A	0.9800
C18—H18B	0.9800	C46—H46B	0.9800
C18—H18C	0.9800	C46—H46C	0.9800
C19—C23	1.410 (4)	C47—C48	1.415 (4)
C19—C20	1.422 (4)	C47—C51	1.421 (4)
C19—C24	1.504 (4)	C47—C52	1.507 (4)
C19—Hf1	2.563 (2)	C47—Hf2	2.563 (3)
C20—C21	1.419 (4)	C48—C49	1.414 (4)
C20—C25	1.501 (4)	C48—C53	1.505 (4)
C20—Hf1	2.571 (3)	C48—Hf2	2.576 (2)
C21—C22	1.423 (4)	C49—C50	1.425 (4)
C21—C26	1.496 (3)	C49—C54	1.490 (4)
C21—Hf1	2.587 (3)	C49—Hf2	2.570 (3)
C22—C23	1.419 (4)	C50—C51	1.422 (4)
C22—C27	1.498 (4)	C50—C55	1.505 (4)
C22—Hf1	2.570 (2)	C50—Hf2	2.544 (3)
C23—C28	1.509 (4)	C51—C56	1.511 (4)
C23—Hf1	2.523 (3)	C51—Hf2	2.580 (2)
C24—H24A	0.9800	C52—H52A	0.9800
C24—H24B	0.9800	C52—H52B	0.9800
C24—H24C	0.9800	C52—H52C	0.9800
C25—H25A	0.9800	C53—H53A	0.9800
C25—H25B	0.9800	C53—H53B	0.9800
C25—H25C	0.9800	C53—H53C	0.9800
C26—H26A	0.9800	C54—H54A	0.9800
C26—H26B	0.9800	C54—H54B	0.9800
C26—H26C	0.9800	C54—H54C	0.9800
C27—H27A	0.9800	C55—H55A	0.9800
C27—H27B	0.9800	C55—H55B	0.9800
C27—H27C	0.9800	C55—H55C	0.9800
C28—H28A	0.9800	C56—H56A	0.9800
C28—H28B	0.9800	C56—H56B	0.9800
C28—H28C	0.9800	C56—H56C	0.9800
			
C2—C1—Hf1	128.05 (18)	C41—C40—C39	108.1 (2)
C2—C1—H1A	105.3	C41—C40—C45	125.6 (3)
Hf1—C1—H1A	105.3	C39—C40—C45	126.0 (3)
C2—C1—H1B	105.3	C41—C40—Hf2	73.46 (14)
Hf1—C1—H1B	105.3	C39—C40—Hf2	75.58 (15)
H1A—C1—H1B	106.0	C45—C40—Hf2	122.48 (19)
C3—C2—C1	113.7 (2)	C40—C41—C37	107.9 (2)
C3—C2—H2A	108.8	C40—C41—C46	123.7 (3)
C1—C2—H2A	108.8	C37—C41—C46	126.6 (3)
C3—C2—H2B	108.8	C40—C41—Hf2	74.22 (15)
C1—C2—H2B	108.8	C37—C41—Hf2	73.51 (14)
H2A—C2—H2B	107.7	C46—C41—Hf2	129.97 (18)
C2—C3—C4	114.4 (3)	C37—C42—H42A	109.5
C2—C3—H3A	108.7	C37—C42—H42B	109.5
C4—C3—H3A	108.7	H42A—C42—H42B	109.5
C2—C3—H3B	108.7	C37—C42—H42C	109.5
C4—C3—H3B	108.7	H42A—C42—H42C	109.5
H3A—C3—H3B	107.6	H42B—C42—H42C	109.5
C3—C4—H4A	109.5	C38—C43—H43A	109.5
C3—C4—H4B	109.5	C38—C43—H43B	109.5
H4A—C4—H4B	109.5	H43A—C43—H43B	109.5
C3—C4—H4C	109.5	C38—C43—H43C	109.5
H4A—C4—H4C	109.5	H43A—C43—H43C	109.5
H4B—C4—H4C	109.5	H43B—C43—H43C	109.5
C6—C5—Hf1	117.75 (17)	C39—C44—H44A	109.5
C6—C5—H5A	107.9	C39—C44—H44B	109.5
Hf1—C5—H5A	107.9	H44A—C44—H44B	109.5
C6—C5—H5B	107.9	C39—C44—H44C	109.5
Hf1—C5—H5B	107.9	H44A—C44—H44C	109.5
H5A—C5—H5B	107.2	H44B—C44—H44C	109.5
C7—C6—C5	112.8 (2)	C40—C45—H45A	109.5
C7—C6—H6A	109.0	C40—C45—H45B	109.5
C5—C6—H6A	109.0	H45A—C45—H45B	109.5
C7—C6—H6B	109.0	C40—C45—H45C	109.5
C5—C6—H6B	109.0	H45A—C45—H45C	109.5
H6A—C6—H6B	107.8	H45B—C45—H45C	109.5
C8—C7—C6	113.6 (2)	C41—C46—H46A	109.5
C8—C7—H7A	108.8	C41—C46—H46B	109.5
C6—C7—H7A	108.8	H46A—C46—H46B	109.5
C8—C7—H7B	108.8	C41—C46—H46C	109.5
C6—C7—H7B	108.8	H46A—C46—H46C	109.5
H7A—C7—H7B	107.7	H46B—C46—H46C	109.5
C7—C8—H8A	109.5	C48—C47—C51	108.1 (2)
C7—C8—H8B	109.5	C48—C47—C52	126.8 (2)
H8A—C8—H8B	109.5	C51—C47—C52	124.3 (2)
C7—C8—H8C	109.5	C48—C47—Hf2	74.54 (14)
H8A—C8—H8C	109.5	C51—C47—Hf2	74.62 (14)
H8B—C8—H8C	109.5	C52—C47—Hf2	125.20 (18)
C13—C9—C10	108.4 (2)	C49—C48—C47	108.5 (2)
C13—C9—C14	123.0 (3)	C49—C48—C53	125.1 (2)
C10—C9—C14	126.7 (3)	C47—C48—C53	125.7 (2)
C13—C9—Hf1	74.90 (15)	C49—C48—Hf2	73.80 (14)
C10—C9—Hf1	73.30 (14)	C47—C48—Hf2	73.49 (14)
C14—C9—Hf1	130.23 (18)	C53—C48—Hf2	126.25 (18)
C11—C10—C9	107.3 (2)	C48—C49—C50	107.7 (3)
C11—C10—C15	124.5 (2)	C48—C49—C54	126.1 (2)
C9—C10—C15	127.5 (3)	C50—C49—C54	124.9 (3)
C11—C10—Hf1	75.53 (15)	C48—C49—Hf2	74.31 (14)
C9—C10—Hf1	74.05 (14)	C50—C49—Hf2	72.84 (14)
C15—C10—Hf1	123.85 (18)	C54—C49—Hf2	128.65 (19)
C12—C11—C10	108.3 (2)	C51—C50—C49	108.0 (2)
C12—C11—C16	124.7 (3)	C51—C50—C55	127.1 (3)
C10—C11—C16	126.0 (2)	C49—C50—C55	124.3 (3)
C12—C11—Hf1	74.36 (13)	C51—C50—Hf2	75.26 (16)
C10—C11—Hf1	72.16 (13)	C49—C50—Hf2	74.81 (15)
C16—C11—Hf1	128.38 (19)	C55—C50—Hf2	123.0 (2)
C11—C12—C13	107.8 (3)	C47—C51—C50	107.7 (2)
C11—C12—C17	124.0 (3)	C47—C51—C56	123.7 (3)
C13—C12—C17	127.9 (3)	C50—C51—C56	126.6 (3)
C11—C12—Hf1	73.76 (13)	C47—C51—Hf2	73.31 (14)
C13—C12—Hf1	73.26 (14)	C50—C51—Hf2	72.52 (16)
C17—C12—Hf1	123.89 (19)	C56—C51—Hf2	132.66 (18)
C9—C13—C12	108.1 (2)	C47—C52—H52A	109.5
C9—C13—C18	125.4 (3)	C47—C52—H52B	109.5
C12—C13—C18	126.1 (3)	H52A—C52—H52B	109.5
C9—C13—Hf1	73.09 (14)	C47—C52—H52C	109.5
C12—C13—Hf1	74.66 (14)	H52A—C52—H52C	109.5
C18—C13—Hf1	124.00 (18)	H52B—C52—H52C	109.5
C9—C14—H14A	109.5	C48—C53—H53A	109.5
C9—C14—H14B	109.5	C48—C53—H53B	109.5
H14A—C14—H14B	109.5	H53A—C53—H53B	109.5
C9—C14—H14C	109.5	C48—C53—H53C	109.5
H14A—C14—H14C	109.5	H53A—C53—H53C	109.5
H14B—C14—H14C	109.5	H53B—C53—H53C	109.5
C10—C15—H15A	109.5	C49—C54—H54A	109.5
C10—C15—H15B	109.5	C49—C54—H54B	109.5
H15A—C15—H15B	109.5	H54A—C54—H54B	109.5
C10—C15—H15C	109.5	C49—C54—H54C	109.5
H15A—C15—H15C	109.5	H54A—C54—H54C	109.5
H15B—C15—H15C	109.5	H54B—C54—H54C	109.5
C11—C16—H16A	109.5	C50—C55—H55A	109.5
C11—C16—H16B	109.5	C50—C55—H55B	109.5
H16A—C16—H16B	109.5	H55A—C55—H55B	109.5
C11—C16—H16C	109.5	C50—C55—H55C	109.5
H16A—C16—H16C	109.5	H55A—C55—H55C	109.5
H16B—C16—H16C	109.5	H55B—C55—H55C	109.5
C12—C17—H17A	109.5	C51—C56—H56A	109.5
C12—C17—H17B	109.5	C51—C56—H56B	109.5
H17A—C17—H17B	109.5	H56A—C56—H56B	109.5
C12—C17—H17C	109.5	C51—C56—H56C	109.5
H17A—C17—H17C	109.5	H56A—C56—H56C	109.5
H17B—C17—H17C	109.5	H56B—C56—H56C	109.5
C13—C18—H18A	109.5	C1—Hf1—C5	93.18 (10)
C13—C18—H18B	109.5	C1—Hf1—C23	132.49 (10)
H18A—C18—H18B	109.5	C5—Hf1—C23	91.99 (10)
C13—C18—H18C	109.5	C1—Hf1—C10	100.34 (9)
H18A—C18—H18C	109.5	C5—Hf1—C10	131.00 (9)
H18B—C18—H18C	109.5	C23—Hf1—C10	111.17 (9)
C23—C19—C20	107.8 (2)	C1—Hf1—C9	130.25 (9)
C23—C19—C24	126.7 (3)	C5—Hf1—C9	108.73 (10)
C20—C19—C24	123.6 (3)	C23—Hf1—C9	91.87 (8)
C23—C19—Hf1	72.32 (16)	C10—Hf1—C9	32.65 (9)
C20—C19—Hf1	74.20 (14)	C1—Hf1—C19	120.14 (10)
C24—C19—Hf1	131.54 (18)	C5—Hf1—C19	124.09 (10)
C21—C20—C19	108.1 (2)	C23—Hf1—C19	32.19 (9)
C21—C20—C25	126.9 (3)	C10—Hf1—C19	88.55 (9)
C19—C20—C25	124.1 (2)	C9—Hf1—C19	83.79 (8)
C21—C20—Hf1	74.69 (15)	C1—Hf1—C13	121.94 (10)
C19—C20—Hf1	73.65 (14)	C5—Hf1—C13	79.26 (9)
C25—C20—Hf1	126.10 (19)	C23—Hf1—C13	105.43 (9)
C20—C21—C22	107.9 (2)	C10—Hf1—C13	53.63 (8)
C20—C21—C26	126.7 (3)	C9—Hf1—C13	32.01 (9)
C22—C21—C26	124.5 (2)	C19—Hf1—C13	110.79 (9)
C20—C21—Hf1	73.37 (15)	C1—Hf1—C22	104.10 (9)
C22—C21—Hf1	73.31 (14)	C5—Hf1—C22	77.24 (9)
C26—C21—Hf1	127.43 (17)	C23—Hf1—C22	32.35 (8)
C23—C22—C21	107.7 (2)	C10—Hf1—C22	141.24 (9)
C23—C22—C27	126.3 (3)	C9—Hf1—C22	123.75 (8)
C21—C22—C27	125.0 (2)	C19—Hf1—C22	53.15 (9)
C23—C22—Hf1	71.99 (14)	C13—Hf1—C22	128.94 (8)
C21—C22—Hf1	74.66 (14)	C1—Hf1—C20	88.24 (9)
C27—C22—Hf1	128.22 (19)	C5—Hf1—C20	128.82 (9)
C19—C23—C22	108.5 (2)	C23—Hf1—C20	53.40 (9)
C19—C23—C28	127.0 (3)	C10—Hf1—C20	98.70 (8)
C22—C23—C28	124.1 (3)	C9—Hf1—C20	108.75 (8)
C19—C23—Hf1	75.49 (16)	C19—Hf1—C20	32.15 (8)
C22—C23—Hf1	75.66 (14)	C13—Hf1—C20	139.86 (9)
C28—C23—Hf1	121.07 (19)	C22—Hf1—C20	53.09 (9)
C19—C24—H24A	109.5	C1—Hf1—C11	77.56 (10)
C19—C24—H24B	109.5	C5—Hf1—C11	109.83 (9)
H24A—C24—H24B	109.5	C23—Hf1—C11	142.83 (10)
C19—C24—H24C	109.5	C10—Hf1—C11	32.31 (10)
H24A—C24—H24C	109.5	C9—Hf1—C11	53.26 (9)
H24B—C24—H24C	109.5	C19—Hf1—C11	119.82 (9)
C20—C25—H25A	109.5	C13—Hf1—C11	53.02 (8)
C20—C25—H25B	109.5	C22—Hf1—C11	172.75 (9)
H25A—C25—H25B	109.5	C20—Hf1—C11	120.31 (8)
C20—C25—H25C	109.5	C1—Hf1—C12	89.86 (9)
H25A—C25—H25C	109.5	C5—Hf1—C12	79.99 (9)
H25B—C25—H25C	109.5	C23—Hf1—C12	137.47 (9)
C21—C26—H26A	109.5	C10—Hf1—C12	53.46 (9)
C21—C26—H26B	109.5	C9—Hf1—C12	53.12 (8)
H26A—C26—H26B	109.5	C19—Hf1—C12	136.66 (9)
C21—C26—H26C	109.5	C13—Hf1—C12	32.09 (9)
H26A—C26—H26C	109.5	C22—Hf1—C12	153.81 (8)
H26B—C26—H26C	109.5	C20—Hf1—C12	151.19 (8)
C22—C27—H27A	109.5	C11—Hf1—C12	31.88 (8)
C22—C27—H27B	109.5	C1—Hf1—C21	79.19 (9)
H27A—C27—H27B	109.5	C5—Hf1—C21	98.39 (9)
C22—C27—H27C	109.5	C23—Hf1—C21	53.34 (8)
H27A—C27—H27C	109.5	C10—Hf1—C21	130.28 (9)
H27B—C27—H27C	109.5	C9—Hf1—C21	136.78 (8)
C23—C28—H28A	109.5	C19—Hf1—C21	53.02 (8)
C23—C28—H28B	109.5	C13—Hf1—C21	158.72 (9)
H28A—C28—H28B	109.5	C22—Hf1—C21	32.03 (8)
C23—C28—H28C	109.5	C20—Hf1—C21	31.93 (8)
H28A—C28—H28C	109.5	C11—Hf1—C21	144.09 (8)
H28B—C28—H28C	109.5	C12—Hf1—C21	168.85 (8)
C30—C29—Hf2	131.33 (18)	C29—Hf2—C33	91.61 (9)
C30—C29—H29A	104.4	C29—Hf2—C37	98.01 (10)
Hf2—C29—H29A	104.4	C33—Hf2—C37	131.48 (9)
C30—C29—H29B	104.4	C29—Hf2—C41	128.90 (9)
Hf2—C29—H29B	104.4	C33—Hf2—C41	112.16 (10)
H29A—C29—H29B	105.6	C37—Hf2—C41	32.66 (9)
C31—C30—C29	112.4 (2)	C29—Hf2—C50	133.69 (9)
C31—C30—H30A	109.1	C33—Hf2—C50	94.00 (10)
C29—C30—H30A	109.1	C37—Hf2—C50	111.71 (10)
C31—C30—H30B	109.1	C41—Hf2—C50	90.70 (9)
C29—C30—H30B	109.1	C29—Hf2—C40	123.36 (10)
H30A—C30—H30B	107.9	C33—Hf2—C40	81.39 (9)
C32—C31—C30	115.2 (3)	C37—Hf2—C40	53.78 (8)
C32—C31—H31A	108.5	C41—Hf2—C40	32.32 (9)
C30—C31—H31A	108.5	C50—Hf2—C40	102.93 (9)
C32—C31—H31B	108.5	C29—Hf2—C47	87.98 (9)
C30—C31—H31B	108.5	C33—Hf2—C47	127.94 (9)
H31A—C31—H31B	107.5	C37—Hf2—C47	99.97 (9)
C31—C32—H32A	109.5	C41—Hf2—C47	107.96 (9)
C31—C32—H32B	109.5	C50—Hf2—C47	53.41 (9)
H32A—C32—H32B	109.5	C40—Hf2—C47	138.75 (9)
C31—C32—H32C	109.5	C29—Hf2—C49	106.03 (9)
H32A—C32—H32C	109.5	C33—Hf2—C49	77.37 (9)
H32B—C32—H32C	109.5	C37—Hf2—C49	142.08 (10)
C34—C33—Hf2	117.53 (17)	C41—Hf2—C49	122.59 (9)
C34—C33—H33A	107.9	C50—Hf2—C49	32.35 (8)
Hf2—C33—H33A	107.9	C40—Hf2—C49	126.36 (9)
C34—C33—H33B	107.9	C47—Hf2—C49	53.13 (9)
Hf2—C33—H33B	107.9	C29—Hf2—C48	80.52 (9)
H33A—C33—H33B	107.2	C33—Hf2—C48	96.82 (9)
C35—C34—C33	115.5 (2)	C37—Hf2—C48	131.63 (8)
C35—C34—H34A	108.4	C41—Hf2—C48	135.67 (9)
C33—C34—H34A	108.4	C50—Hf2—C48	53.17 (9)
C35—C34—H34B	108.4	C40—Hf2—C48	156.01 (9)
C33—C34—H34B	108.4	C47—Hf2—C48	31.96 (8)
H34A—C34—H34B	107.5	C49—Hf2—C48	31.89 (8)
C36—C35—C34	113.2 (3)	C29—Hf2—C51	119.57 (9)
C36—C35—H35A	108.9	C33—Hf2—C51	125.89 (10)
C34—C35—H35A	108.9	C37—Hf2—C51	89.54 (9)
C36—C35—H35B	108.9	C41—Hf2—C51	82.81 (8)
C34—C35—H35B	108.9	C50—Hf2—C51	32.22 (9)
H35A—C35—H35B	107.8	C40—Hf2—C51	109.15 (9)
C35—C36—H36A	109.5	C47—Hf2—C51	32.07 (8)
C35—C36—H36B	109.5	C49—Hf2—C51	53.15 (9)
H36A—C36—H36B	109.5	C48—Hf2—C51	52.86 (9)
C35—C36—H36C	109.5	C29—Hf2—C39	91.27 (9)
H36A—C36—H36C	109.5	C33—Hf2—C39	79.24 (9)
H36B—C36—H36C	109.5	C37—Hf2—C39	53.24 (9)
C38—C37—C41	107.8 (2)	C41—Hf2—C39	53.20 (8)
C38—C37—C42	124.8 (3)	C50—Hf2—C39	134.93 (9)
C41—C37—C42	127.0 (3)	C40—Hf2—C39	32.12 (9)
C38—C37—Hf2	76.13 (15)	C47—Hf2—C39	152.81 (9)
C41—C37—Hf2	73.82 (14)	C49—Hf2—C39	151.13 (9)
C42—C37—Hf2	122.37 (18)	C48—Hf2—C39	170.84 (9)
C39—C38—C37	107.9 (2)	C51—Hf2—C39	135.97 (8)
C39—C38—C43	123.9 (3)	C29—Hf2—C38	76.90 (9)
C37—C38—C43	127.0 (3)	C33—Hf2—C38	107.64 (9)
C39—C38—Hf2	74.07 (15)	C37—Hf2—C38	32.26 (10)
C37—C38—Hf2	71.61 (13)	C41—Hf2—C38	53.35 (9)
C43—C38—Hf2	129.46 (18)	C50—Hf2—C38	142.73 (9)
C38—C39—C40	108.2 (2)	C40—Hf2—C38	53.11 (9)
C38—C39—C44	123.9 (3)	C47—Hf2—C38	122.76 (8)
C40—C39—C44	127.4 (3)	C49—Hf2—C38	174.29 (9)
C38—C39—Hf2	74.25 (14)	C48—Hf2—C38	146.89 (8)
C40—C39—Hf2	72.30 (15)	C51—Hf2—C38	121.16 (9)
C44—C39—Hf2	125.08 (19)	C39—Hf2—C38	31.68 (9)

## References

[bb1] Bruker (2008). *APEX2*, *SAINT* and *SADABS*. Bruker AXS Inc., Madison, Wisconsin, USA.

[bb2] Burlakov, V. V., Beweries, T., Bogdanov, V. S., Arndt, P., Baumann, W., Petrovskii, P. V., Spannenberg, A., Lyssenko, K. A., Shur, V. B. & Rosenthal, U. (2009). *Organometallics*, **28**, 2864–2870.

[bb3] Burlakov, V. V., Bogdanov, V. S., Lyssenko, K. A., Petrovskii, P. V., Beweries, T., Arndt, P., Rosenthal, U. & Shur, V. B. (2008). *Russ. Chem. Bull.* **57**, 1319–1320.

[bb4] Calhorda, M. J., Carrondo, M. A. A. F. de C. T., Dias, A. R., Galvão, A. M., Garcia, M. H., Martins, A. M., Minas da Piedade, M. E., Pinheiro, C. I., Romão, C. C., Simões, J. A. M. & Veiros, L. F. (1991). *Organometallics*, **10**, 483–494.

[bb5] Ernst, R. D., Harvey, B. G. & Arif, A. M. (2004). *Z. Kristallogr. New Cryst. Struct.*, **219**, 398–400.

[bb6] Flack, H. D. (1983). *Acta Cryst.* A**39**, 876–881.

[bb7] Paolucci, G., Pojana, G., Zanon, J., Lucchini, V. & Avtomonov, E. (1997). *Organometallics*, **16**, 5312–5320.

[bb8] Schock, L. E. & Marks, T. J. (1988). *J. Am. Chem. Soc.* **110**, 7701–7715.

[bb9] Sheldrick, G. M. (2008). *Acta Cryst.* A**64**, 112–122. 10.1107/S0108767307043930 18156677

