# Supplementary figures and images for: Crystal structure of di-n-but­yl­bis­(η 5-penta­methyl­cyclo­penta­dien­yl)hafnium(IV)
Source: Acta Crystallogr E Crystallogr Commun. 2015 Jan 10;71(Pt 2):m19–20. doi: 10.1107/S2056989015000092 (PMC4384584; doi:10.1107/S2056989015000092)

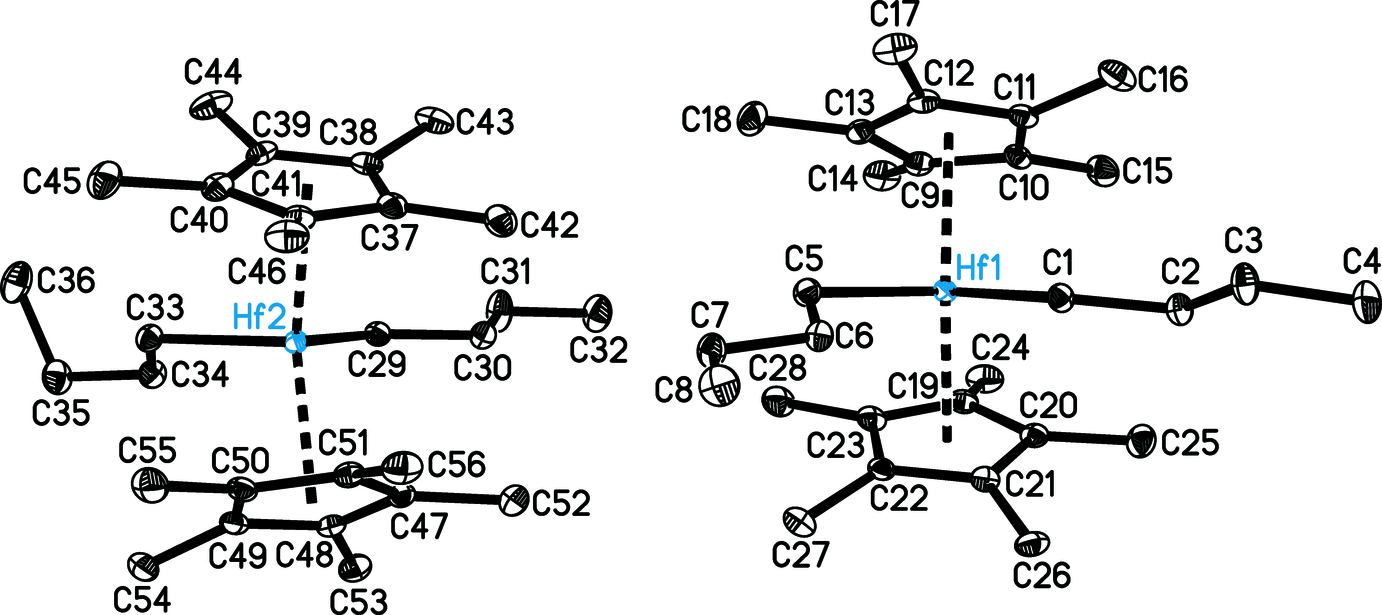

Supplement: Supplementary file 3 [file e-71-00m19-fig1.tif]
